# Chemoenzymatic Radiosynthesis of a Gluconate Transporter‐Targeted In Vivo Bacterial Sensor From Clinical [^18^F]FDG

**DOI:** 10.1002/anie.8729932

**Published:** 2026-08-11

**Authors:** Sang Hee Lee, Jung Min Kim, Joseph Blecha, Robert R. Flavell, Michael A. Ohliger, Joanne Engel, Youngho Seo, David M. Wilson

**Affiliations:** ^1^ Department of Radiology and Biomedical Imaging University of California San Francisco California USA; ^2^ UCSF Helen Diller Family Comprehensive Cancer Center University of California San Francisco California USA; ^3^ Department of Pharmaceutical Chemistry University of California San Francisco California USA; ^4^ Department of Radiology Zuckerberg San Francisco General Hospital San Francisco California USA; ^5^ Department of Medicine University of California San Francisco California USA; ^6^ Department of Nuclear Engineering University of California Berkeley California USA

**Keywords:** [^18^F]FDG, chemoenzymatic radiosynthesis, gluconate metabolism, glucose oxidase, positron emission tomography

## Abstract

Many bacteria, but not mammalian cells, can directly import and metabolize extracellular gluconate via gluconate permease (GntP) and kinase (GntK). We hypothesized that fluorine‐18 labeled gluconic acid ([^18^F]FGA) could leverage unique bacterial gluconate metabolism for pathogen‐specific PET imaging. Here, we report [^18^F]FGA as a new PET tracer targeting bacterial gluconate metabolism. The chemoenzymatic radiosynthesis of [^18^F]FGA was simple, only requiring readily available [^18^F]FDG and glucose oxidase. [^18^F]FGA showed broad detection sensitivity across multiple bacterial species, including multidrug‐resistant clinical isolates. *Staphylococcus aureus* transposon mutant studies showed substantial loss of [^18^F]FGA uptake in GntP and GntK mutants, supporting high specificity for bacterial gluconate metabolism. [^18^F]FGA PET selectively highlighted live bacterial infection, with high target‐to‐nontarget ratios in *S. aureus* (36‐fold) and *Escherichia coli* (30‐fold) in a murine model of myositis. Unlike [^18^F]FDG, no appreciable uptake of [^18^F]FGA was detected at sites of sterile inflammation. In a *Klebsiella pneumoniae* pneumonia model, [^18^F]FGA showed significantly higher accumulation in the infected lung (5.2 ± 0.8%IA/cc) than in the uninfected lung (0.1 ± 0.0%IA/cc), further supporting its potential for imaging challenging clinical infections. Taken together, [^18^F]FGA PET may be a useful tool for pathogen‐targeted imaging in clinical practice.

## Graphical abstract

An efficient radiosynthesis of fluorine‐18 labeled gluconic acid ([^18^F]FGA) was developed using two readily available materials, 2‐deoxy‐2‐[^18^F]fluoro‐D‐glucose ([^18^F]FDG) and glucose oxidase. [^18^F]FGA is highly specific for bacterial gluconate metabolism mediated by gluconate permease (GntP) and gluconate kinase (GntK), with rapid clearance, low off‐target signals, and selective detection of live bacterial infection over sterile inflammation.

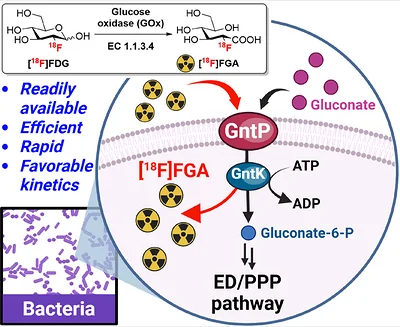

## 1 Introduction

Rapid and specific diagnosis of bacterial infections remains a major clinical challenge, especially given increasing antimicrobial resistance. A recent systematic analysis estimated that antimicrobial resistance associated deaths increased from 2000 to 2021, with *Escherichia coli*, *Klebsiella pneumoniae*, and *Staphylococcus aureus* representing an increasing proportion of causative pathogens [1]. While the recent development of new antibiotics is promising, many bacterial pathogens have already evolved resistance to them. Moreover, antibiotic overuse increases antimicrobial resistance and contributes to the emergence of “superbugs”. In this context, identifying the true bacterial burden at an early stage of infection is critical for guiding appropriate therapy and improving infection management [2].

Early identification of bacterial infection remains challenging because current imaging modalities (e.g., computed tomography and magnetic resonance imaging) primarily rely on secondary structural changes associated with infection and inflammation and cannot distinguish between the two. Recent positron emission tomography (PET) imaging approaches targeting human pathogens aim to differentiate live bacteria from mammalian cells [3]. Over the past decade, numerous imaging strategies have taken advantage of bacteria‐specific metabolic pathways, including amino acid incorporation and carbohydrate metabolism [4, 5], folate biosynthesis [6, 7], and siderophore‐mediated iron transport [8]. However, there is no stand‐alone imaging agent for routine differentiation of infection from sterile inflammation in clinical practice, as many candidate PET tracers are limited by short half‐life of carbon‐11 (20.38 min), insufficient specificity, synthetic hurdles, or limited availability of key materials.

To date, 2‐deoxy‐2‐[^18^F]fluoro‐D‐sorbitol ([^18^F]FDS) is one of the most practical infection tracers, particularly for imaging *Enterobacteriaceae* [9]. Radiosynthesis of [^18^F]FDS from widely available 2‐deoxy‐2‐[^18^F]fluoro‐D‐glucose ([^18^F]FDG) is straightforward by chemical reduction using sodium borohydride [10, 11]. Together with its low off‐target signals, the synthetic ease of [^18^F]FDS has contributed to its broad accessibility, facilitating tracer use worldwide. In this context, the use of the widely available PET synthon [^18^F]FDG could potentially enable “on‐demand” production of a PET radiotracer. This concept is well suited to chemoenzymatic radiosynthesis, in which [^18^F]FDG serves as an acceptor in enzymatic glycosylation to generate fluorine‐18 labeled disaccharides (Figure 1A) [12, 13, 14, 15]. In 2001, the first chemoenzymatic radiosynthesis of ^18^F‐lactose was reported for imaging the expression of the *lacZ* gene [16]. This approach was later applied to ^18^F‐trehalose synthesis via trehalose synthase (TreT) for imaging *Mycobacteria* [14, 15]. More recently, our group further expanded this strategy by establishing a phosphorylase‐based platform for generating fluorine‐18 labeled disaccharides via reverse phosphorolysis using α/β‐glucose‐1‐phosphate as a donor and [^18^F]FDG as an acceptor [12]. Collectively, these studies highlight the versatility of [^18^F]FDG as a readily available ^18^F synthon for chemical and chemoenzymatic diversification into pathogen‐specific imaging radiotracers. Motivated by recent translational needs as well as the versatility of [^18^F]FDG, we sought to develop a more practical infection‐targeted radiotracer using a simple enzymatic transformation with a common and inexpensive enzyme.

**FIGURE 1:**
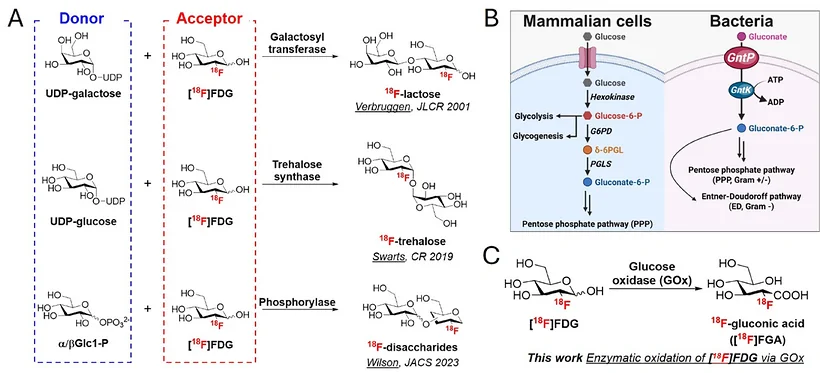
(A) Representative chemoenzymatic radiosynthesis of ^18^F‐disaccharides from [^18^F]FDG. UDP‐galactose, UDP‐glucose, and α/βGlc‐1‐P were used as donors, and [^18^F]FDG served as the acceptor. The reactions were catalyzed by galactosyltransferase, trehalose synthase, or phosphorylase enzymes to generate the corresponding ^18^F‐labeled disaccharides. (B) Schematic illustration of mammalian and bacterial gluconate metabolism. In mammalian cells, extracellular glucose is transported into the cell and phosphorylated to glucose‐6‐phosphate, which is subsequently converted to gluconate‐6‐phosphate via glucose‐6‐phosphate dehydrogenase (G6PD) and 6‐phosphogluconolactonase (PGLS). In bacteria, extracellular gluconate can be transported by GntP and subsequently phosphorylated by GntK to gluconate‐6‐phosphate. Gluconate‐6‐phosphate enters the pentose phosphate pathway or the Entner–Doudoroff pathway. (C) Scheme for the chemoenzymatic radiosynthesis of ^18^F‐gluconic acid ([^18^F]FGA) reported in this study. Readily available GOx from *Aspergillus niger* (EC 1.1.3.4) enables the direct conversion of clinical [^18^F]FDG to [^18^F]FGA without the need for additional reagents.

Glucose oxidase (GOx) is an enzyme found in fungi and insects, but not in mammals [17]. GOx catalyzes the oxidation of glucose to gluconolactone, which is then spontaneously hydrolyzed to gluconic acid, while hydrogen peroxide is generated as a byproduct and contributes to antibacterial and antifungal activity [18]. GOx is found in many organisms, while the enzyme from *Aspergillus niger* is the most widely available and its properties, including substrate specificity and kinetics, have been well characterized [18, 19]. Interestingly, although all deoxy‐D‐glucose analogs showed lower specificity versus D‐glucose, 2‐deoxy‐D‐glucose was a better substrate (25%–30% activity relative to glucose) than f 3‐, 4‐, or 6‐deoxy‐D‐glucose (1%–10%) [19]. Inspired by these findings, we hypothesized that [^18^F]FDG might also serve as a substrate for GOx to generate 2‐deoxy‐2‐fluoro‐D‐gluconic acid ([^18^F]FGA).

6‐phosphogluconic acid is a key metabolic intermediate in central carbon metabolism. The pentose phosphate pathway is well conserved across prokaryotes and eukaryotes [20], whereas the Entner–Doudoroff pathway is more restricted to prokaryotes [21]. Among bacteria, the Entner–Doudoroff pathway is found in some gram‐negative species including those from the *Enterobacteriaceae* family but is rare in Gram‐positive bacteria [22, 23, 24]. In mammalian cells, extracellular glucose is imported and converted into 6‐phosphogluconate (Figure 1B). In bacteria, extracellular glucose can be transported through the bacterial phosphotransferase system (PTS), which is distinct from mammalian glucose transporters, and subsequently metabolized to 6‐phosphogluconate [25]. In addition, unlike mammalian cells, many bacteria can directly import extracellular gluconate through GntP, followed by phosphorylation via GntK [26, 27]. We therefore sought to exploit differences in gluconate metabolism between bacteria and mammalian cells for pathogen‐specific imaging. By leveraging the bacterial gluconate metabolic pathway, we hypothesized that [^18^F]FGA could detect bacterial infection with low off‐target signals in mammalian tissues.

Here, we developed a chemoenzymatic radiosynthesis of fluorine‐18 labeled gluconic acid ([^18^F]FGA) from widely available [^18^F]FDG for targeting bacterial gluconate metabolism (Figure 1C). [^18^F]FGA was synthesized by GOx‐mediated oxidation of [^18^F]FDG without additional reagents, followed by simple cartridge‐based purification using NH_2_ and Alumina Sep‐Pak. We validated the bacterial specificity of [^18^F]FGA across multiple bacterial species, including multidrug‐resistant clinical isolates in vitro. We further investigated the uptake of [^18^F]FGA in *S. aureus* transposon mutants to probe the relevant metabolic pathway. Next, we characterized [^18^F]FGA in vivo in uninfected mice, in a lipopolysaccharide (LPS) induced myositis model, and in bacterial infection models. Together, these studies support the potential of [^18^F]FGA as a sensitive and specific PET tracer for detecting bacterial infections.

## 2 Results and Discussion

### 2.1 Radiosynthesis of [^18^F]FGA Using Clinical [^18^F]FDG and GOx

We examined whether FDG could serve as a substrate for GOx by reacting nonradioactive FDG (50 mg) with GOx (5 mg) at 35°C for 4 h. The resulting product, nonradioactive FGA, was expected to be retained more strongly on the Plus NH_2_ Sep Pak cartridge than FDG, whereas FDG could be efficiently removed by 95% MeCN/water based on our established protocol. Under the same purification protocol that we previously established for [^18^F]FSK [28], Sep Pak purification of the reaction mixture under these conditions afforded FGA with high chemical purity, and neither FDG nor the intermediate fluorinated gluconolactone was detected by mass (Figure S1) or NMR analysis (Figure S2). In the ^19^F composite pulse decoupling (CPD) analysis, the glucose analog FDG showed two distinct signals corresponding to the alpha (δ −199.34 ppm) and beta (δ −199.48 ppm) anomers (Figure S3). In contrast, a sharp singlet was observed downfield after oxidation (δ −196.68 ppm), clearly indicating the ring‐opening of FDG via GOx‐mediated oxidation. The molecular structure was confirmed via 2D ^1^H‐^13^C heteronuclear single quantum coherence (HSQC) NMR analysis. In the ^1^H proton NMR spectrum of FGA, the C2 proton (δ 4.87 ppm) appeared as a doublet of doublets (dd) with a geminal ^1^H‐^19^F coupling constant (^2^ *J*_H‐F_ = 50.0 Hz) and a vicinal ^1^H‐^1^H coupling to the C3 proton (^3^ *J*_H‐H_ = 3.7 Hz). The 2D ^1^H‐^13^C HSQC spectrum indicated that the C2 proton was correlated to the C2 carbon (δ 92.18 ppm), which appeared as a doublet with a direct ^13^C‐^19^F coupling constant (^1^ *J*_C‐F_ = 185.5 Hz). Subsequently, the C3 proton (δ 4.09 ppm) appeared as a doublet of triplets (dt) with a vicinal ^1^H‐^19^F coupling (^3^ *J*_H‐F_ = 24.4 Hz) and ^1^H‐^1^H coupling to the C2 and C4 proton (^3^ *J*_H‐H_ = 3.3 Hz), which was correlated to the C3 carbon (δ 70.40 ppm; a doublet with a geminal ^13^C‐^19^F constant, ^2^ *J*_C‐F_ = 18.8 Hz). The C4 and C5 protons appeared as a multiplet between δ 3.73–3.65 ppm and were correlated to the C4 (δ 71.50 ppm) and C5 carbons (δ 71.12 ppm). The C6 protons appeared as an ABX system in the ranges of δ 3.81–3.73 ppm and δ 3.64–3.54 ppm, which was correlated to the singlet C6 carbon (δ 62.67 ppm). The C1 carbon appeared as a doublet with a geminal ^13^C‐^19^F constant (^2^ *J*_C‐F_ = 21.3 Hz), no correlation in the ^1^H‐^13^C HSQC spectrum due to the absence of a proton at the carbonyl carbon (Figure S2).

Encouraged by these preliminary results on the mass scale, we next applied this strategy to the radiosynthesis of [^18^F]FGA from a clinical [^18^F]FDG solution (Figure 2A). Clinical [^18^F]FDG received from our cyclotron (36.3 ± 10.2 mCi in 0.5 mL, produced using an [^18^F]FDG citrate cassette on the GE FASTlab module from up to 6 Ci [^18^F]fluoride ion) was reacted with GOx (1 mg) under vigorous stirring. Radio‐HPLC analysis of the crude reaction mixture indicated the formation of [^18^F]FGA (*T*_R_ = 12 min), whereas unreacted [^18^F]FDG was observed at approximately 5 min, with no appreciable side product seen. Under the same purification protocol, the product was eluted with saline (3 mL) to afford formulated [^18^F]FGA with a 42.5 ± 6.4% decay‐corrected radiochemical yield (*n* = 16; 10.6 ± 3.4 mCi). No detectable residual [^18^F]FDG was observed in the final formulated of [^18^F]FGA (Figure 2B). Total synthesis time was about 60 min. The radiochemical identity of [^18^F]FGA was confirmed by co‐injection with a nonradioactive FGA standard (Figure 2C). The in vitro stability of [^18^F]FGA was evaluated in the formulation solution (saline), mouse serum, and human serum at 37°C for up to 4 h using analytical HPLC. [^18^F]FGA remained intact over 4 h under all tested conditions (Figure 2D).

**FIGURE 2:**
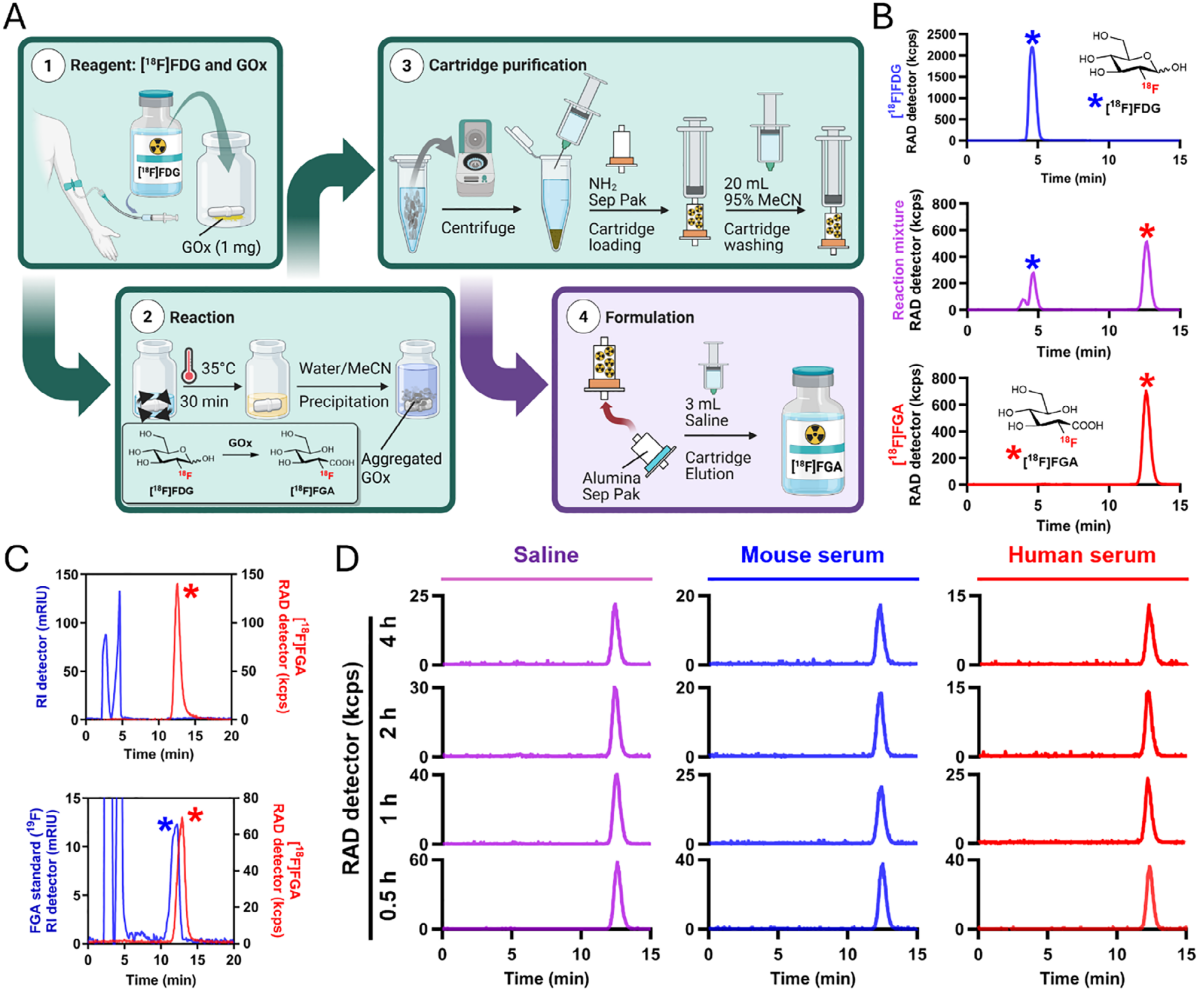
GOx‐mediated chemoenzymatic radiosynthesis of [^18^F]FGA from clinical [^18^F]FDG. (A) Schematic illustration of the radiosynthesis, cartridge purification, and formulation workflow for [^18^F]FGA. Clinical [^18^F]FDG solution was added to a vial containing GOx (1 mg). The mixture was reacted at 35°C for 30 min under vigorous stirring. After the reaction, the enzyme was precipitated by the addition of 80% MeCN/water, followed by centrifugation. The resulting supernatant was further diluted with MeCN, loaded onto a NH_2_ Sep Pak, and washed with 20 mL of 95% MeCN. A dried NH_2_ Sep Pak was then connected to an alumina Sep Pak, and the product was eluted with saline (3 mL). (B) Representative radio‐HPLC profile of [^18^F]FDG and [^18^F]FGA using an Amaze‐TH column (4.6 × 150 mm, 5 µm, 75% MeCN with 20 mM acetate, flow rate: 1 mL/min). The blue and red asterisks indicate [^18^F]FDG and [^18^F]FGA, respectively. (C) Radio‐HPLC analysis for confirmation of the radiochemical identity of [^18^F]FGA, without (top) and with (bottom) a nonradioactive FGA standard. (D) In vitro stability analysis of [^18^F]FGA in saline, mouse, and human serum at 37°C after 0.5, 1, 2, and 4 h incubation.

We demonstrated that a manual radiosynthesis protocol could provide sufficient [^18^F]FGA for preclinical studies, and potentially for a single patient dose (typically 5–15 mCi for an ^18^F tracer). A fully automated radiosynthesis of [^18^F]FGA will be further optimized for clinical translation. Recently, automated chemoenzymatic radiosyntheses of the [^18^F]FDG‐derived disaccharides [^18^F]FDC and [^18^F]FDT were reported using up to 100 mCi [^18^F]FDG reacted with enzyme (1.5 mg of cellobiose phosphorylase for [^18^F]FDC; 1 mg of OtsA and OtSB, 5 mg of hexokinase for [^18^F]FDT) [13, 15]. We anticipate that chemoenzymatic radiosynthesis within < 3 half‐lives of [^18^F]FDG production would be most feasible to implement in practice, while the reaction performed later (> 5 half‐lives) from the time of [^18^F]FDG production might affect conversion efficiency due to increased volume and glucose content. To address this concern, glucose scavenging techniques might offer substantial benefits to chemoenzymatic radiosyntheses using clinical [^18^F]FDG, including the GOx‐mediated oxidation in this report. We additionally investigated the effect of GOx amount on radiochemical conversion yields (RCC) of [^18^F]FDG to [^18^F]FGA to aid future automation. The RCC increased with the amount of GOx from 0.1 to 4 mg, with nearly quantitative conversion achieved (<2% residual [^18^F]FDG) using 4 mg of GOx under vigorous stirring at 35°C for 30 min (Figure S4). The chemoenzymatic reaction will be further optimized by studying various parameters including the addition of oxygen, reaction time, volume, amount of GOx, and glucose content.

### 2.2 In Vitro Uptake of [^18^F]FGA in Diverse Bacteria

[^18^F]FDS was reported as an *Enterobacteriaceae*‐selective tracer that exploits the sorbitol metabolism pathway associated with the *srlD* gene [29]. More recent studies have shown that [^18^F]FDS is also taken up by clinically relevant fungal pathogens, including *Aspergillus fumigatus*, *Rhizopus arrhizus*, and *Candida albicans*, and has been evaluated in patients with invasive mold infections [30, 31]. However, the bacterial specificity of [^18^F]FDS is largely restricted to the *Enterobacteriaceae* family, including *E. coli*, *Enterobacter cloacae*, and *K. pneumoniae*, due to the absence of *srlD* in many other species [32].

As a preliminary study, we examined the distribution of putative GntP homologs across bacterial species using protein‐protein BLAST to predict which bacterial species might take up [^18^F]FGA. GntP is highly conserved among *Staphylococcus* species, whereas no putative GntP homologs were identified in *Streptococcus* species (*S. agalactiae, S. pyogenes, and S. pneumoniae*) (Table S1). Consistent with previous reports, no putative GntP homologs were identified in *Listeria monocytogenes*, whereas GntP in *Bacillus subtilis* showed 62.42% sequence identity to *S. aureus* [33]. *Enterococcus faecalis* showed low sequence identity of GntP to *S. aureus*. *E. coli* has more complicated gluconate metabolism, including a high‐affinity GntP (encoded by *gntT*) and a low‐affinity GntP (encoded by *gntU*) [34]. These two GntP homologs are also highly conserved across *Enterobacteriaceae* family (*E. coli*, *Salmonella* species, *E. cloacae*, and *K. pneumoniae*; > 93% sequence identity to *E. coli*). Only one GntP homolog was identified in *Acinetobacter baumannii*, *Pseudomonas aeruginosa*, and *Proteus mirabilis*. *P. mirabilis* showed relatively high sequence identity to the low‐affinity GntP of *E. coli* (76.6%), whereas *A. baumannii* and *P. aeruginosa* showed low sequence identity to both high‐ and low‐affinity GntP of *E. coli* (< 50%) (Tables S2 and S3).

Given that putative GntP homologs were identified in a subset of gram‐positive and negative bacteria, we next explored uptake of [^18^F]FGA in *S. aureus*, an important cause of human infections. In vitro uptake of [^18^F]FGA in *S. aureus* increased over time, with no statistically significant difference between 90 and 120 min (*p* = 0.3086) (Figure 3A). To assess the bacterial specificity of [^18^F]FGA uptake, we performed in vitro assays under different incubation conditions (Figure 3B). [^18^F]FGA uptake in *S. aureus* was markedly inhibited by co‐incubation with either 1 mM of gluconic acid ([^18^F]FGA+GA) or a nonradioactive FGA standard ([^18^F]FGA+FGA). A sulfhydryl‐blocking reagent, *p*‐chloromercuribenzoate (PCMB), also dramatically reduced the uptake of [^18^F]FGA, supporting a transporter‐mediated uptake rather than passive diffusion [35]. A competition uptake assay in *S. aureus* demonstrated that [^18^F]FGA uptake decreased in the presence of unlabeled gluconic acid above 40 µg/mL, suggesting the involvement of a specific transport system (Figure 3C) [29]. No appreciable uptake of [^18^F]FGA was observed in heat‐killed *S. aureus*. These data suggest that [^18^F]FGA uptake in *S. aureus* is specific and may be closely related to bacterial gluconate metabolism.

**FIGURE 3:**
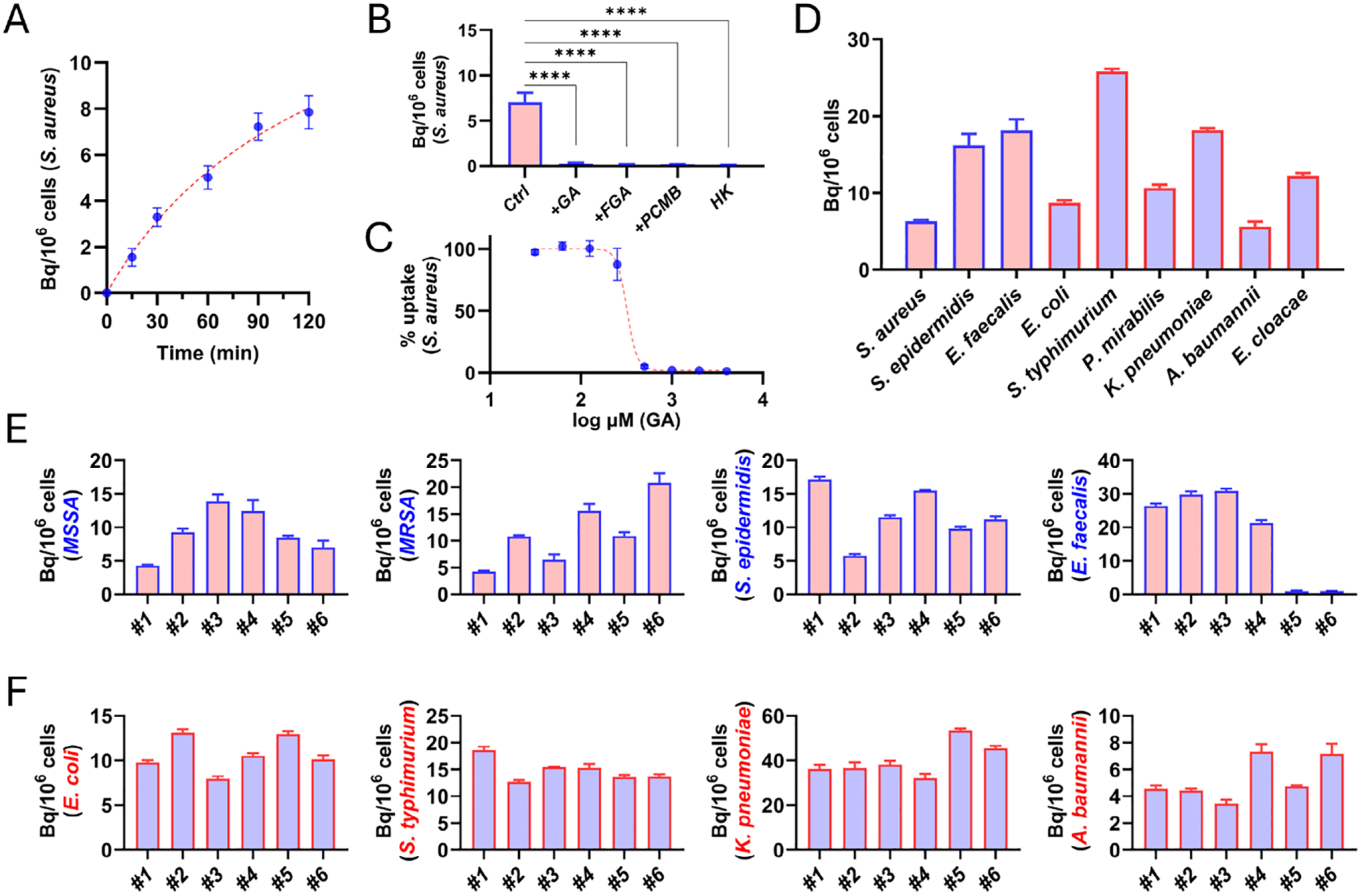
In vitro evaluation of [^18^F]FGA uptake in bacteria, including clinical isolates. (A) Time‐course in vitro uptake of [^18^F]FGA in *S. aureus*. The uptake of [^18^F]FGA increased over time during incubation. (B) In vitro uptake of [^18^F]FGA in *S. aureus* under different incubation conditions. GA: gluconic acid, FGA: ^19^F‐standard of [^18^F]FGA, PCMB. Data were analyzed by one‐way ANOVA with Dunnett's multiple comparisons test; ****P<0.0001. (C) [^18^F]FGA uptake with increasing concentration of gluconic acid in *S. aureus*. The uptake was normalized to that without the addition of gluconate. (D) Screening of [^18^F]FGA uptake in a panel of bacteria. Blue outlines indicate Gram‐positive bacteria, and red outlines indicate Gram‐negative bacteria. (E, F) In vitro uptake assay of [^18^F]FGA across multiple clinical isolates of Gram‐positive (E) and Gram‐negative (F) bacteria.

To explore the scope of bacterial uptake and to determine if [^18^F]FGA uptake correlates with the presence of these homologs, we screened [^18^F]FGA uptake across a panel of candidate bacterial species in vitro (Figure 3D). [^18^F]FGA showed broad bacterial uptake, including *S. aureus*, *E. coli*, *K. pneumoniae*, *A. baumannii*, and *Salmonella typhimurium*, which were listed in WHO bacterial priority pathogens in 2024 [36]. Uptake specificity was further confirmed using heat‐killed bacteria (Figure S5). The [^18^F]FGA uptake in *Streptococcus species* (*S. agalactiae* and *S. pyogenes*) and *L. monocytogenes* was negligible, with no significant difference between live and heat‐killed bacteria (Figure S6). The [^18^F]FGA uptake in *P. aeruginosa* was minimal but specific, which was demonstrated using blocking (1 mM gluconic acid) and heat‐killed controls (*p* < 0.0001). *P. aeruginosa* has a Gntp gene (PA2322) [37], which is known to be induced upon growth in either glucose or gluconate [38]. In our study, [^18^F]FGA uptake was not affected by growth on 30 mM glucose (*p* = 0.5534), while growth on 30 mM gluconate showed a subtle increase in uptake (*p* = 0.0104) (Figure S7). We hypothesize that the observed minimal accumulation may be due to low substrate affinity for the 2‐deoxy analog. However, future studies are required for further verification.

To further probe the impact of fluorination position on bacterial uptake, we examined in vitro uptake of 4‐deoxy‐4‐[^18^F]fluoro‐D‐gluconic acid (4‐[^18^F]FGA), which is the constitutional isomer of [^18^F]FGA (C2 substituted analog). Surprisingly, 4‐[^18^F]FGA uptake was observed only in *Staphylococcus* species (*S. aureus* and *S. epidermidis*), indicating that the position of fluorine substitution strongly influences bacterial target specificity. Another constitutional isomer of [^18^F]FDS, 4‐deoxy‐4‐[^18^F]fluoro‐D‐sorbitol (4‐[^18^F]FDS), was not taken up by all bacteria we tested (Figure S8). Protein‐protein BLAST analysis showed that the protein sequence of GntP is highly conserved across *Staphylococcus* species (> 86% identity to *S. aureus*), whereas gram‐negative bacteria, as well as *E. faecalis*, showed much lower sequence identity to *S. aureus* (< 53%) (Table S4). These findings suggest that loss of the C4 hydroxyl group has a minor effect on recognition by the GntP of *Staphylococcus* species but may be critical for recognition by GntPs from other bacterial species. Although loss of the C4 hydroxyl group greatly limits the range of bacterial detection capacity, 4‐[^18^F]FGA may be a useful constitutional isomer of [^18^F]FGA as an *S. aureus*‐specific sensor.

We next tested the uptake of [^18^F]FGA across clinical bacteria isolates, including multidrug‐resistant (MDR) bacterial strains. In gram‐positive clinical isolates, [^18^F]FGA showed specific uptake in clinical isolates of methicillin‐sensitive *S. aureus* (MSSA), methicillin‐resistant *S. aureus* (MRSA), and vancomycin‐intermediate *S. epidermidis*. Meanwhile, two MDR *E. faecalis* strains showed negligible uptake (Figure 3E). The observed strain‐to‐strain variability in [^18^F]FGA uptake may reflect strain‐dependent heterogeneity in gluconate metabolism. Future studies investigating the expression profiles of GntP and GntK across various clinical isolates are needed to fully elucidate the correlation between their expression levels and tracer uptake. Uptake of [^18^F]FGA was observed in all tested MDR gram‐negative strains (Figure 3F). Taken as a whole, these data support the broad detection sensitivity of [^18^F]FGA across clinically relevant bacteria including MDR pathogens. The bacterial strains used in this study are listed in Tables S5 and S6.

### 2.3 Biodistribution and Dosimetry Analysis of [^18^F]FGA in Uninfected Mice

[^18^F]FDG is the most widely used PET tracer in nuclear medicine for imaging abnormal glucose metabolism [39]. However, [^18^F]FDG PET lacks sensitivity for infection, since it primarily relies on the host inflammatory response, which could be blunted in immunosuppressed patients [40]. In addition, [^18^F]FDG uptake cannot differentiate bacterial infection from sterile inflammation, including autoimmune diseases and post‐surgical scarring, leading to false‐positive interpretations [41]. Moreover, [^18^F]FDG PET is dependent on imaging conditions and has organ‐specific limitations, complicating the interpretation of imaging data [40]. Therefore, an ideal PET radiotracer for imaging infectious diseases requires (1) high specificity for bacteria and can distinguish infection from host inflammatory processes, (2) high target‐to‐nontarget signal with low off‐target signals, and (3) robustness to variation in imaging conditions and physiological states.

To assess in vivo distribution and elimination profile of [^18^F]FGA, PET data were acquired on a 90 min list mode immediately after tracer administration in uninfected mice (*n* = 4, unconscious). Representative time‐course PET images indicate rapid distribution and clearance of [^18^F]FGA at early time after tracer injection, followed by rapid urinary excretion (Figure 4A). In a different mice cohort, we performed a 5 min static scan of [^18^F]FGA at 85 min after tracer injection to assess if physiological states affect in vivo profile of tracer (Figure 4A). Unlike [^18^F]FDG PET, which is highly affected by the imaging conditions [42], [^18^F]FGA PET showed comparable biodistribution under different physiological conditions (conscious vs unconscious) in both in vivo and *ex vivo* analysis, with only minimal differences likely attributable to slower blood circulation under the unconscious state (Figure 4B,C).

**FIGURE 4:**
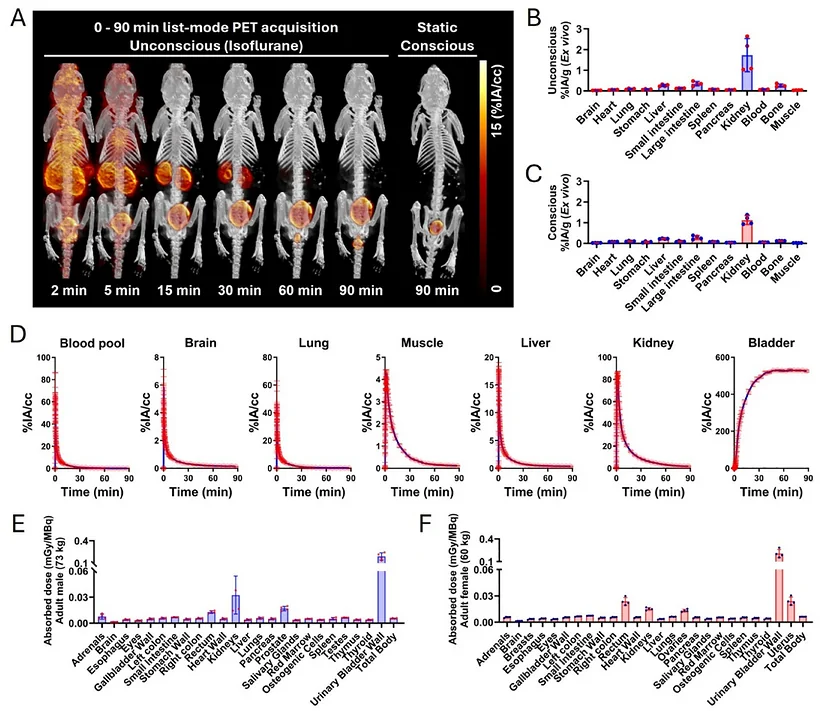
Biodistribution and dosimetry analysis of [^18^F]FGA in uninfected mice. (A) Representative time‐course images (unconscious) and static image (conscious) of [^18^F]FGA in uninfected mice (*n* = 4 for each). “Conscious” indicates mice that recovered from anesthesia after tracer injection to allow normal physical activity before a static scan. (B, C) *Ex vivo* biodistribution of [^18^F]FGA in the unconscious (B) and conscious (C) state. (D) Time‐activity curves were generated from list‐mode PET acquisition data and presented as %IA/cc (mean ± SEM). (E, F) Estimated human absorbed dose in organs extrapolated from male (E) and female (F) murine biodistribution data using ICRP publication 103 tissue weighting factors.

The pharmacokinetic parameters of [^18^F]FGA were derived from the time‐activity curves (Figure 4D) and summarized in Table 1. The calculated plasma elimination half‐life (T_1/2β_) was 10.42 min, which is shorter than that of [^18^F]FDS (18.74 min) reported in our previous study [43]. [^18^F]FGA showed peak uptake (*C*_max_) in the blood pool and major organs (brain, lung, and liver) at early time after injection (*T*_max_ < 1 min). The kidneys showed higher peak uptake (*C*_max_ = 90.6 ± 3.6%IA/cc) and the value of area under the curve (AUC_0–90 min_ = 977.3 ± 120.6%IA/cc·min) than that of liver (*C*_max_ = 18.7 ± 1.9%IA/cc, *AUC*_0–90 min_ = 100.7 ± 8.5%IA/cc·min), indicating dominant renal clearance of [^18^F]FGA with urinary excretion. Notably, the value of *C*_max_ and AUC_0–90 min_ of [^18^F]FGA in liver is significantly lower than that of [^18^F]FDS (*C*_max_ = 28.6 ± 1.9%IA/cc, AUC_0–90 min_ = 289.4 ± 21.3%IA/cc·min). Together with dominant urinary clearance, the overall short elimination half‐life in major organs, including the kidneys, suggest that [^18^F]FGA PET may also have potential utility as a renal functional imaging agent [44]. Muscle showed relatively low peak uptake (*C*_max_ = 4.7 ± 0.4%IA/cc, *T*_max_ = 1.3 ± 0.6 min), followed by rapid washout (*T*_1/2β_ = 19.62 min, AUC_0–90 min_ = 57.9 ± 6.9%IA/cc·min). [^18^F]FGA radioactivity in muscle decreased to below 0.15 ± 0.03%IA/cc at 60 min post‐injection, which is lower than the reported value for [^18^F]FDS and [^18^F]FMtl (^18^F‐mannitol; C2 epimer of [^18^F]FDS), highlighting the low soft‐tissue background of [^18^F]FGA PET [5, 45]. Taken as a whole, these data indicate that [^18^F]FGA exhibits favorable pharmacokinetic properties for infection imaging, including rapid clearance and low off‐target signals.

**TABLE 1:** Time‐activity curve derived pharmacokinetic parameters of [^18^F]FGA in uninfected mice under the unconscious state (*n* = 4). IA: Injected activity; *C*_max_: Maximum concentration; *T*_max_: Time to maximum concentration; AUC: Value of area under the curve; T_1/2α_: Distribution half‐life; T_1/2β_: Elimination half‐life.

Parameters	Blood pool	Brain	Lung	Muscle	Liver	Kidneys	Bladder
*C*_max_ (%IA/cc)	87.5 ± 6.9	7.3 ± 0.5	61.3 ± 16.5	4.7 ± 0.4	18.7 ± 1.9	90.6 ± 3.6	538.4 ± 4.7
*T*_max_ (min)	0.2 ± 0.1	0.3 ± 0.1	0.2 ± 0.1	1.3 ± 0.6	0.4 ± 0.1	1.4 ± 0.4	65.0 ± 6.5
AUC (%IA/cc·min)	187.9 ± 19.4	30.5 ± 4.0	164.7 ± 23.0	57.9 ± 6.9	100.7 ± 8.5	977.3 ± 120.6	40350.0 ± 1214.2
*T*_1/2α_ (min)	0.81	1.37	0.79	3.84	1.50	n.d.	n.d.
*T*_1/2β_ (min)	10.42	22.81	10.67	19.62	24.01	21.71	n.d.

We further estimated human radiation doses by extrapolating murine (*n* = 4 each) biodistribution data to human using ICRP publication 60 and 103 tissue weighting factors (Figure 4E,F). All organs exhibited lower absorbed doses than the reported value for [^18^F]FDG [46], except the kidneys and urinary bladder wall. Manual bladder voiding was not performed in this study, which likely contributed to the higher absorbed dose to the urinary bladder wall. The estimated effective doses in both male (0.012 mSv/MBq) and female (0.014 mSv/MBq) were comparable with common ^18^F‐radiotracers ([^18^F]FDG: 0.017 mSv/MBq, [^18^F]FLT: 0.015 mSv/MBq, and [^18^F]choline: 0.011 mSv/MBq) [47, 48]. Complete dosimetry profiles with ICRP publication 60 tissue weighting factors are summarized in Tables S7 and S8.

### 2.4 Metabolic Specificity of [^18^F]FGA for the Gluconate Transport System in *S. aureus*

Building upon our preliminary in vitro and in vivo findings, we next investigated the specificity of [^18^F]FGA for bacterial gluconate metabolism. Given the structural similarity between gluconate and [^18^F]FGA, we hypothesized that [^18^F]FGA may be utilized through the gluconate metabolic pathway in *S. aureus*. This hypothesis was further supported by the blocking studies with nonradioactive gluconate and FGA shown in Figure 3B,C. The gluconate transport and phosphorylation system of *S. aureus* was identified: GntP (encoded by *gntP*; SAUSA300_2442) and GntK (encoded by *gntK*; SAUSA300_2443) [49]. To investigate the uptake pathway, we compared in vitro uptake of [^18^F]FGA in a *S. aureus* JE2 strain and two isogenic JE2 transposon mutants (Δ*gntP* and Δ*gntK*). Fluorine‐18 labeled maltose ([^18^F]FDM) was selected as a control tracer, as our previous transposon mutant study demonstrated that its uptake is mediated by the maltose/maltodextrin transport system (*malE*, *malF*, *malG*, and *malK*) (Figure 5A) [12]. We observed no appreciable tracer uptake in the Δ*gntP* mutant, suggesting that [^18^F]FGA uptake in *S. aureus* requires GntP (Figure 5B). [^18^F]FGA uptake was significantly reduced in the Δ*gntK* mutant, suggesting that intracellular phosphorylation via GntK may contribute to tracer retention. The uptake of [^18^F]FDM was not affected by disruption of *gntP* or *gntK*, suggesting that the gluconate metabolic pathway is specific for [^18^F]FGA in *S. aureus*.

**FIGURE 5:**
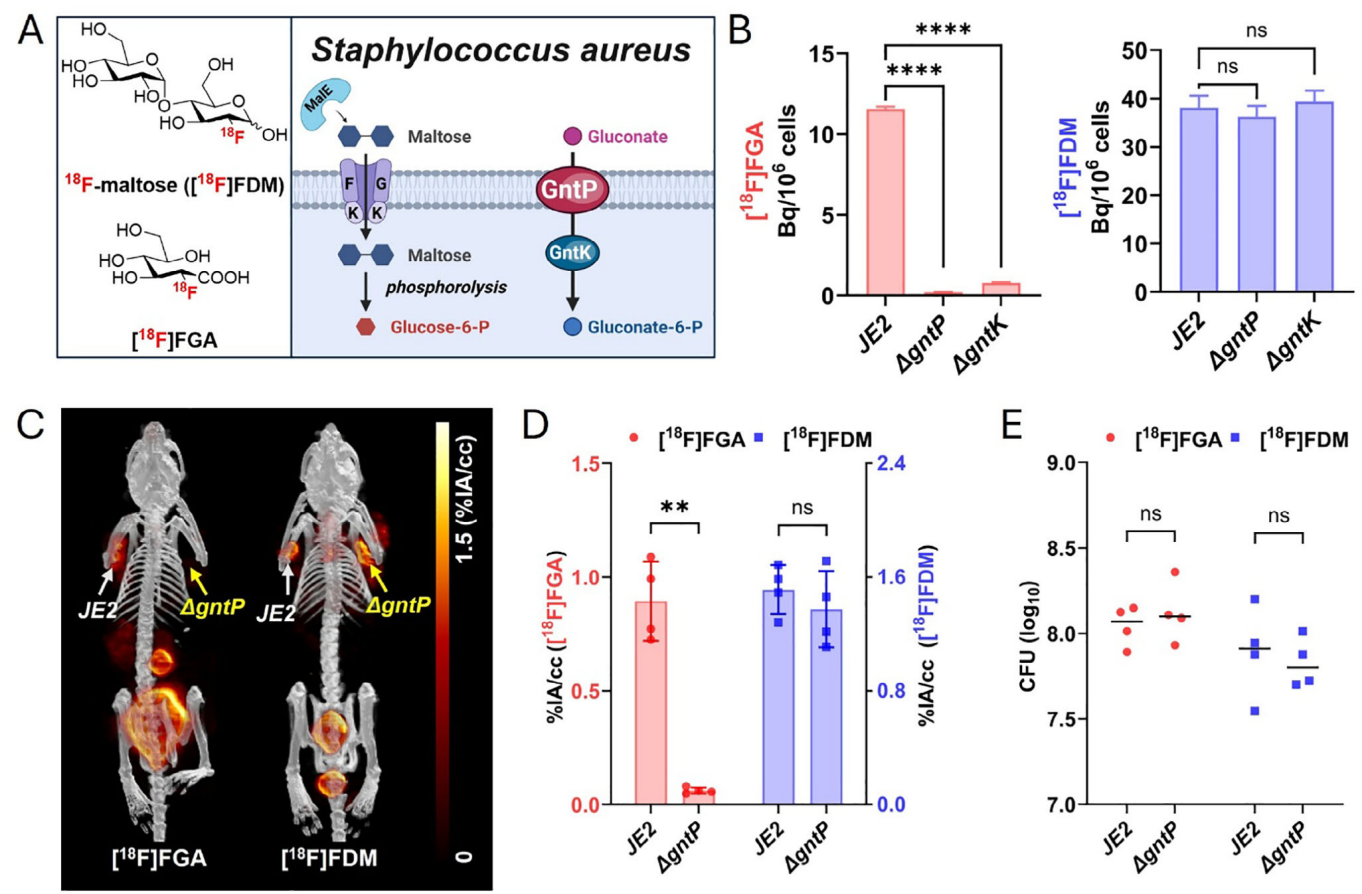
Characterization of [^18^F]FGA uptake in *S. aureus* via the bacterial gluconate transport pathway. (A) Structures of ^18^F‐maltose ([^18^F]FDM) and [^18^F]FGA, and a schematic illustration of maltose and gluconate metabolism in *S. aureus*. [^18^F]FDM was used as a control tracer for comparison. (B) In vitro uptake assay of [^18^F]FGA and [^18^F]FDM in *S. aureus* transposon mutants (Δ*gntP* and Δ*gntK*) and their parental strain JE2. (C) Specificity of [^18^F]FGA PET for bacterial gluconate metabolism in a *S. aureus* transposon mutant myositis model (*n* = 4). Voglibose (1 mg per mouse) was co‐injected with [^18^F]FDM to inhibit α‐glucosidase activity. (D) in vivo and *ex vivo* quantification analysis data of [^18^F]FGA and [^18^F]FDM in JE2/Δ*gntP* mutant myositis mice. (E) Bacterial counts in homogenized tissues (infected triceps muscles). Data were analyzed using a one‐way ANOVA (in vitro) or a paired Student's *t*‐test (in vivo/*ex vivo*); ns, not significant; **P<0.01 and ****P<0.0001.

We further tested the use of [^18^F]FGA PET in a murine model of myositis (Figure 5C). Mice were infected with *S. aureus* JE2 strain in the right triceps muscle and Δ*gntP* mutant in the left triceps muscle. [^18^F]FGA PET was conducted without any metabolic inhibitor, whereas [^18^F]FDM PET was conducted by co‐injection with voglibose (α‐glucosidase inhibitor, 1 mg per head) to prevent in vivo degradation [12]. [^18^F]FGA showed 15.2 ± 4.3 fold higher uptake at the JE2 infected muscle (0.90 ± 0.17%IA/cc) than Δ*gntP* mutant infected muscle (0.06 ± 0.01%IA/cc, *p* = 0.0026). The radioactivity of [^18^F]FGA PET at the site of Δ*gntP* mutant infection was not significantly different with that in uninfected mice (*p* = 0.4250). In contrast, uptake of [^18^F]FDM was not significantly different between JE2 and Δ*gntP* mutant infected muscles (1.51 ± 0.17 vs 1.37 ± 0.27%IA/cc, *p* = 0.2996) (Figure 5D). *Ex vivo* CFU quantification data confirmed comparable bacterial burdens between JE2 and Δ*gntP* mutant, further supporting that the observed uptake of [^18^F]FGA in *S. aureus* JE2 strain were not driven by difference in bacterial burden or host inflammation (Figure 5E). Taken as a whole, these data support both specificity and selectivity of [^18^F]FGA for gluconate‐dependent metabolism in active *S. aureus*.

### 2.5 Evaluation of [^18^F]FGA PET in Murine Myositis and Pulmonary Infection Models

We further evaluated [^18^F]FGA PET in murine myositis models (*n* = 4 each) to assess its ability to distinguish bacterial infection from sterile inflammation. Infection was induced by intramuscular inoculation of live *S. aureus* or *E. coli* (10^6^ cells) into the left triceps muscle, with a 10‐fold higher concentration of heat‐killed bacteria injected into the contralateral muscle, 6 h before imaging study. For sterile inflammation, LPS (5 mg/kg) was injected into the left triceps muscle 96 h prior to imaging study. At 80 min post injection of [^18^F]FGA, PET data were acquired using a 10 min static scan. In the LPS‐induced myositis model, no significant [^18^F]FGA uptake was observed at the site of LPS injection (0.06 ± 0.02%IA/cc), similar to that observed in the uninfected mice (0.06 ± 0.01%IA/cc) (Figure 6A). Quantitative analyses of the left triceps muscle showed no significant difference between uninfected mice and LPS‐treated mice both in vivo (*p* = 0.9470) and *ex vivo* (*p* = 0.7777) (Figure 6B). In contrast, [^18^F]FDG PET showed 4.1 ± 0.7 fold higher activity at the site of LPS injected muscle (9.5 ± 2.0%IA/cc; *p* = 0.004) than in the contralateral triceps muscle (2.3 ± 0.4%IA/cc) (Figure 6A). In bacterial myositis models, [^18^F]FGA showed focal uptake in muscles infected with live *S. aureus* (2.5 ± 0.4%IA/cc) and *E. coli* (1.9 ± 0.3%IA/cc), whereas negligible uptake was observed in muscles injected with heat‐killed bacteria (heat‐killed *S. aureus*: 0.07 ± 0.01%IA/cc, heat‐killed *E. coli*: 0.07 ± 0.01%IA/cc). The ROI‐derived target‐to‐nontarget ratios (Live vs HK) were markedly higher for *S. aureus* (36.4 ± 4.6) and *E. coli* (29.6 ± 7.3) than for LPS‐treated mice (1.1 ± 0.2) (Figure 6C,D). To better understand the in vivo kinetics of [^18^F]FGA at the site of bacterial infection, we conducted a dynamic PET analysis of [^18^F]FGA in a *S. aureus* myositis model (Figure S9). [^18^F]FGA exhibited rapid accumulation at both live and heat‐killed bacteria inoculated sites during the early distribution phase (< 5 min). Subsequently, the radioactivity of [^18^F]FGA was rapidly cleared from the heat‐killed site, while the activity at the live *S. aureus* inoculated site gradually increased over time (Figure S9A,B). Accordingly, the T/NT ratios of live to heat‐killed and live to blood pool at 110 min (mid time) post injection were 47.7 ± 11.2 and 37.7 ± 8.9, respectively, (Figure S9C,D). These values were not significantly different from those obtained at 70 and 90 min (Figure S9E,F).

**FIGURE 6:**
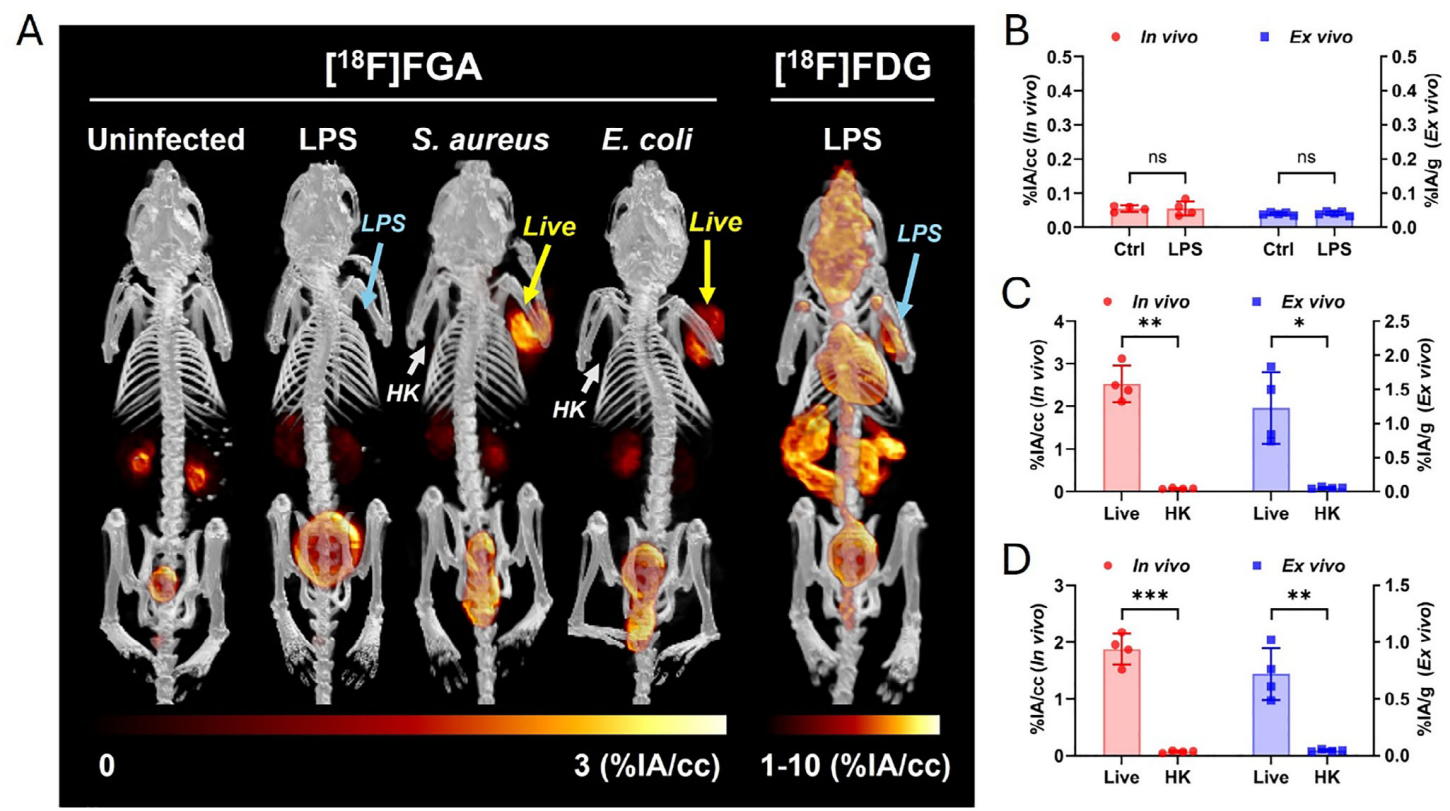
Evaluation of [^18^F]FGA PET in murine myositis models (*n* = 4 each). (A) Representative MIP images of [^18^F]FGA in uninfected mice (Ctrl) and murine myositis models (LPS, *S. aureus*, and *E. coli*). [^18^F]FDG was used as a reference tracer in the LPS‐induced myositis model. Light blue arrows indicate the site of LPS injection. Yellow and white arrows indicate the sites of inoculation with live and heat‐killed bacteria, respectively. (B–D) in vivo and *ex vivo* quantification analysis data of [^18^F]FGA in LPS (B), *S. aureus* (C), and *E. coli* (D) myositis models. Data were analyzed using a paired Student's *t*‐test; ns, not significant; *P<0.05; **P<0.01; ***P<0.001.

Next, given the short plasma elimination half‐life and low nonspecific uptake of [^18^F]FGA in the lung (Table 1), we anticipated that [^18^F]FGA PET would be suitable for imaging pulmonary infections, in which interpretation could be confounded by blood pool background associated with pulmonary perfusion. Pulmonary infection was induced by intratracheal inoculation of *K. pneumoniae* (10^6^ cells). A 10 min [^18^F]FGA static scan was performed 80 min after tracer administration. In a *K. pneumoniae* pulmonary infection model, [^18^F]FGA showed significantly higher tracer accumulation in the infected lung (5.16 ± 0.80%IA/cc) than in the uninfected lung (0.09 ± 0.02%IA/cc). Representative whole‐body MIP image showed a focal [^18^F]FGA signal in the lung (Red arrows). Co‐registered µPET/CT images indicated that the focal [^18^F]FGA uptake localized to the region of lung infiltration identified on CT (Yellow arrows) (Figure 7A). Quantitative analyses indicated significantly higher [^18^F]FGA uptake in the infected lung than in the uninfected lung both in vivo (*p* = 0.001) and *ex vivo* (*p* = 0.0035) (Figure 7B).

**FIGURE 7:**
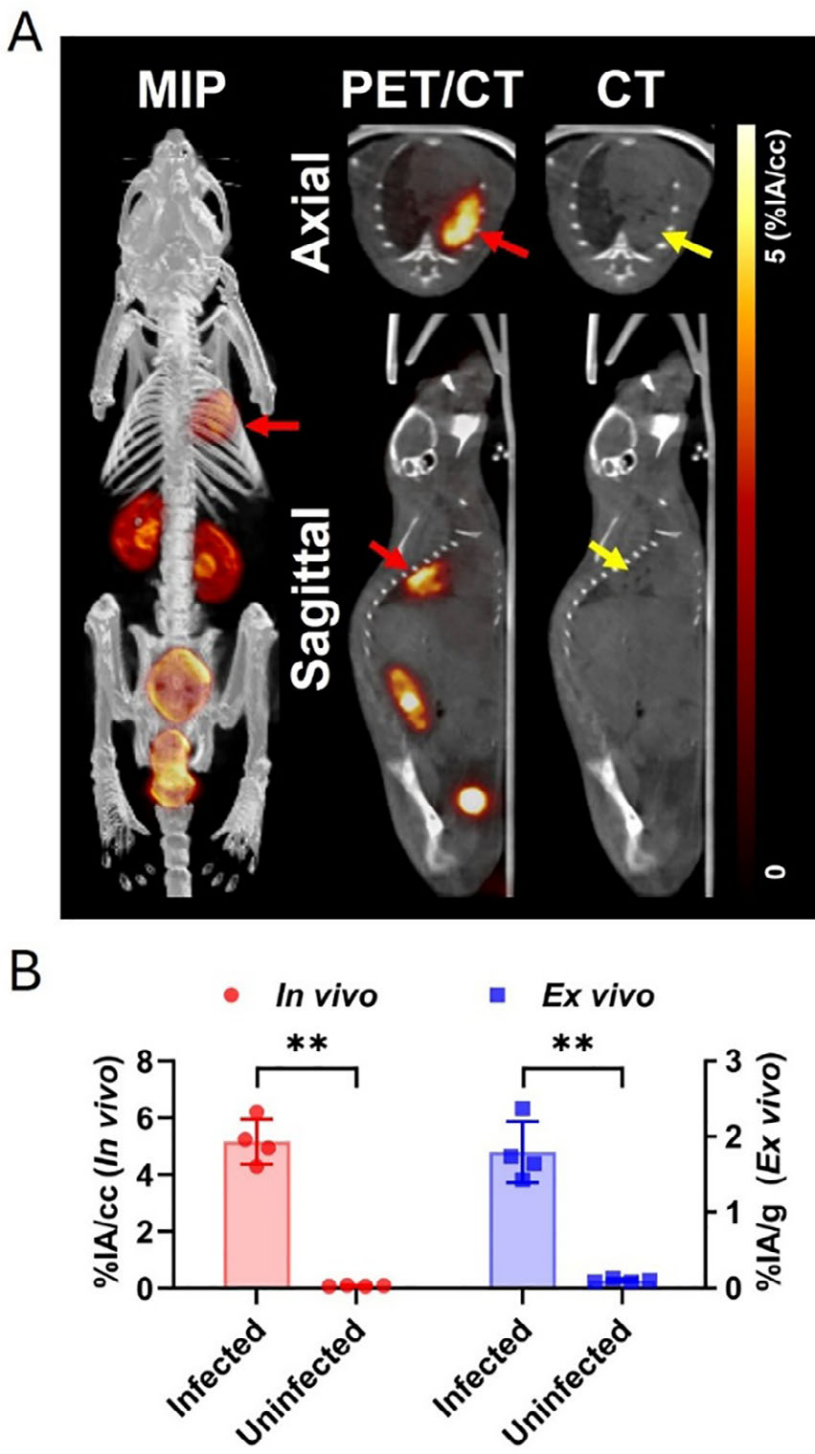
Evaluation of [^18^F]FGA PET in a *K. pneumoniae* pulmonary infection model (*n* = 4). (A) Representative MIP, PET/CT, and CT images of [^18^F]FGA in a *K. pneumoniae* pulmonary infection model. Red arrows indicate the site of *K. pneumoniae* inoculation which is colocalized with the region of lung infiltration (yellow arrows) identified on CT. (B) In vivo and *ex vivo* quantification analysis, compared with uninfected mice (Ctrl). Data were analyzed using an unpaired Student's *t*‐test; **P<0.01.

Collectively, these results suggest that [^18^F]FGA PET can distinguish live bacterial infection from sterile inflammation with minimal off‐target signals, providing high‐contrast imaging of infected sites. While the current study focuses on the discovery and validation of [^18^F]FGA in straightforward preclinical models, the next steps are to investigate [^18^F]FGA PET for monitoring longitudinal antibiotic responses as well as determine its detection sensitivity prior to clinical translation. Furthermore, evaluating [^18^F]FGA in fungal pathogens represents an important future direction, as GntK has been identified in various pathogenic fungi, such as *Cryptococcus*, *Aspergillus*, and *Candida* species [50], and plays an essential role in utilizing gluconate as a carbon source [51, 52]. It is also worth noting that bacteria‐targeted metabolic tracers, such as [^18^F]FDS, [^18^F]FMtl, and [^18^F]FGA, leverage bacterial metabolic pathways that exist in both pathogenic and nonpathogenic bacterial strains. Indeed, our in vitro evaluation indicated a relatively low but specific uptake of [^18^F]FGA in the nonpathogenic probiotic strain *Escherichia coli* Nissle 1917 (Figure S10). Consequently, signals in tissues highly enriched with commensals, such as the intestine and caecum, should be interpreted with caution, as is the case for most PET tracers targeting bacterial metabolism. Despite potentially limited gastrointestinal absorption [53], future investigations utilizing oral administration of [^18^F]FGA may provide insights on the tracer interactions with the gut microbiota in vivo. Lastly, it is important to consider a broader challenge shared among [^18^F]FDG‐derived radiotracers, including [^18^F]FDS (sorbitol) [9], [^18^F]FDC (cellobiose) [13], [^18^F]FDT (trehalose) [14], [^18^F]FDM (maltose) [28], and [^18^F]FSK (nigerose) [28] in addition to [^18^F]FGA (gluconic acid) reported in this study. Clinical [^18^F]FDG samples contain a significant amount of glucose, which is also a substrate for the chemical and biochemical transformations reported. Tracers derived from [^18^F]FDG therefore contain significant amounts of unlabeled native sugars/sugar‐derived metabolites. These unlabeled metabolites lower the effective molar activity of their ^18^F‐labeled correlates and may affect the PET signals observed, although this effect has not been systematically investigated. The effective molar activity of [^18^F]FDG‐derived tracers can be increased using glucose‐scavenging techniques [54] that may both facilitate chemoenzymatic radiosyntheses and improve target accumulation in vivo.

## 3 Conclusion

In conclusion, we developed a facile chemoenzymatic radiosynthesis of [^18^F]FGA for imaging bacterial gluconate metabolism. The use of widely available [^18^F]FDG may enable cyclotron‐independent implementation of [^18^F]FGA PET at sites that can receive [^18^F]FDG, but lack the infrastructure for full de novo production starting from [^18^F]fluoride ion. Moreover, the use of two readily available materials, [^18^F]FDG and GOx, supports a more practical and accessible workflow for broader implementation of [^18^F]FGA PET. Importantly, [^18^F]FGA demonstrated rapid clearance with low off‐target signals in uninfected tissues and showed high specificity for differentiating live bacterial infection from sterile inflammation. In vitro and in vivo transposon mutant studies also underscore the specificity of [^18^F]FGA PET for bacterial gluconate metabolism. Future studies will explore immobilized GOx platforms for cartridge‐based and flow‐through synthesis of [^18^F]FGA, with the goal of enabling a simple and streamlined workflow.

## Author Contributions

**Sang Hee Lee**: conceptualization, investigation, writing – original draft, formal analysis, methodology, software. **Jung Min Kim**: investigation, methodology. **Joseph Blecha**: investigation, methodology. **Robert R. Flavell**: investigation, writing – review and editing. **Michael A. Ohliger**: investigation, writing – review and editing, funding acquisition. **Joanne Engel**: investigation, writing – review and editing. **Youngho Seo**: investigation, methodology, writing – review and editing, software. **David M. Wilson**: conceptualization, investigation, funding acquisition, writing – original draft, methodology.

## Conflicts of Interest

The authors declare no conflicts of interest.

## Supporting information




**Supporting File**: The authors have cited additional references within the Supporting Information [12, 17, 55].

## Data Availability

The data that support the findings of this study are available from the corresponding author upon reasonable request.
