# Supplementary material for: Chemoenzymatic Radiosynthesis of a Gluconate Transporter‐Targeted In Vivo Bacterial Sensor From Clinical [18F]FDG
Source: Angew Chem Int Ed Engl. 2026 Aug 11;65(41):e8729932. doi: 10.1002/anie.8729932 (PMC13638095; doi:10.1002/anie.8729932)
Supplement: Supplementary file 1 [file ANIE-65-e8729932-s001.docx]

**Supporting Information for**

**Title: Chemoenzymatic radiosynthesis of a gluconate transporter-targeted *in vivo* bacterial sensor from clinical [^18^F]FDG**

Sang Hee Lee^[a]^, Jung Min Kim^[a]^, Joseph Blecha^[a]^, Robert R. Flavell^[a,b,c]^, Michael A. Ohliger^[a,d]^, Joanne Engel^[e]^, Youngho Seo^[a,b,f]^, David M. Wilson^[a]^*

^a^Department of Radiology and Biomedical Imaging, University of California, San Francisco, San Francisco, CA 94158, United States.

^b^UCSF Helen Diller Family Comprehensive Cancer Center, University of California, San Francisco, San Francisco, CA 94158, United States

^c^Department of Pharmaceutical Chemistry, University of California, San Francisco, San Francisco, CA 94158, United States

^d^Department of Radiology, Zuckerberg San Francisco General Hospital, San Francisco, CA 94110, United States.

^e^Department of Medicine, University of California, San Francisco, San Francisco, CA 94143, United States.

^f^Department of Nuclear Engineering, University of California, Berkeley, Berkeley, CA 94720, United States.

**Contents:**

**Supplemental Figure and Table**	page 2 - 22

Figure S1. Mass spectrometric analysis of non-radioactive FGA after Sep Pak purification.

Figure S2. NMR analysis of standard FGA in D_2_O.

Figure S3. ^19^F NMR spectrum of 2-deoxy-2-fluoro-D-glucose (FDG).

Figure S4. Further optimization of GOx-mediated [^18^F]FDG oxidation.

Figure S5. *In vitro* evaluation of [^18^F]FGA uptake in a panel of live and heat-killed bacteria.

Figure S6. *In vitro* comparative uptake of [^18^F]FGA in live and heat-killed bacteria.

Figure S7. *In vitro* uptake of [^18^F]FGA in *P. aeruginosa* (PAO1) under different incubation conditions.

Figure S8. Synthesis and bacterial uptake of 4-[^18^F]FDS and 4-[^18^F]FGA.

Figure S9. Dynamic μPET/CT scans of [^18^F]FGA in a *S. aureus* myositis model.

Figure S10. *In vitro* uptake of [^18^F]FGA in *E. coli* (ATCC 25922) and *E. coli* Nissle 1917.

Table S1. Protein-protein BLAST analysis of *S. aureus* GntP Homology in gram-positive bacteria.

Table S2. Protein-protein BLAST analysis of *E. coli* high-affinity GntP Homology in gram-negative bacteria.

Table S3. Protein-protein BLAST analysis of *E. coli* low-affinity GntP Homology in gram-negative bacteria.

Table S4. Protein-protein BLAST analysis of *S. aureus* GntP Homology in gram-negative bacteria.

Table S5. Bacterial strains used in this study.
Table S6. Clinical bacterial strains used in this study.

Table S7. Estimated absorbed dose (mGy/MBq) data extrapolated from female mice data using ICRP60.

Table S8. Estimated absorbed dose (mGy/MBq) data extrapolated from male mice data using ICRP60.

**Materials and Methods**	page 22 - 26

**Figure S1**. Mass spectrometric analysis of non-radioactive FGA after Sep Pak purification. Signals observed at m/z 197.0467, 198.0501, and 199.0509 for non-radioactive FGA. No mass corresponding to residual FDG (m/z 182.0591) or the intermediate fluorinated gluconolactone (m/z 180.0343) was detected.

**Figure S2.** NMR analysis of standard FGA in D_2_O.

**2-deoxy-2-fluoro-gluconic acid** ^1^H NMR

**2-deoxy-2-fluoro-gluconic acid** ^13^C NMR

**2-deoxy-2-fluoro-gluconic acid** ^19^F NMR

**2-deoxy-2-fluoro-gluconic acid** HSQC NMR

**Figure S3**. ^19^F NMR spectrum of 2-deoxy-2-fluoro-D-glucose (FDG). FDG showed two ^19^F signals at δ -199.34 ppm and δ -199.48 ppm, corresponding to its alpha and beta anomers, respectively.

**Figure S4.** Further optimization of GOx-mediated [^18^F]FDG oxidation. (A) Representative radio HPLC profiles (Amaze-TH column; 4.6×250 mm, 10 μm, 75% MeCN with 20 mM acetate, flow rate: 3 mL/min) of the reaction with GOx ranging from 0.1 to 4.0 mg. The black asterisk indicates the [^18^F]FDG peak, while the purple asterisk indicates the [^18^F]FGA peak. (B) Comparative radiochemical conversion (RCC, %) at different GOx amounts (n = 3 for each). Nearly quantitative conversion of [^18^F]FDG to [^18^F]FGA was achieved using 4.0 mg of GOx at 35°C for 30 min.

**Figure S5**. *In vitro* evaluation of [^18^F]FGA uptake in a panel of live and heat-killed bacteria (n = 3). Data were analyzed by multiple unpaired t-test with Welch’s correction.

**Figure S6**. *In vitro* comparative uptake of [^18^F]FGA in live and heat-killed bacteria (n = 3). Data were analyzed by multiple unpaired t-test.

**Figure S7**. *In vitro* uptake of [^18^F]FGA in *P. aeruginosa* (PAO1) under different incubation conditions (n = 3 for each). Blocking: co-incubation with 1 mM gluconate. HK: heat-killed *P. aeruginosa*. Condition A: *P. aeruginosa* (PAO1) were cultured overnight in LB broth containing 30 mM glucose. Condition B: *P. aeruginosa* (PAO1) were cultured overnight in LB broth containing 30 mM gluconate. Cells were collected by centrifugation, washed with PBS, and resuspended in fresh LB broth prior to *in vitro* uptake experiment. Data were analyzed by one-way ANOVA with Dunnett’s multiple comparisons test; *****P* < 0.0001.

**Figure S8**. Synthesis and bacterial uptake of 4-[^18^F]FDS and 4-[^18^F]FGA. (A) Synthesis of 4-fluorinated analog, 4-[^18^F]FDS and 4-[^18^F]FGA from 4-[^18^F]FDG (C4-deoxy) via NaBH_4_-mediated reduction and GOx-mediated oxidation, respectively. (B) *In vitro* uptake assay of 4-[^18^F]FDS and 4-[^18^F]FGA across a panel of candidate bacterial species.

**Figure S9**. Dynamic μPET/CT scans of [^18^F]FGA in a *S. aureus* myositis model (n = 4). (A) Representative time-course MIP images of [^18^F]FGA in a *S. aureus* myositis model. (B) Time-activity curve of [^18^F]FGA activity at the live *S. aureus* inoculated and heat-killed *S. aureus* inoculated site. (C-D) ROI-derived ratios of [^18^F]FGA activity of the live/heat-killed (C) and live/blood pool or heat-killed/blood pool (D) over time. (E-F) comparison of ROI-derived ratios of [^18^F]FGA activity of the live/heat-killed (E) and live/blood pool (F) at 70, 90, and 110 min (mid time), respectively.

**Figure S10**. *In vitro* uptake of [^18^F]FGA in *E. coli* (ATCC 25922) and *E. coli* Nissle 1917 (EcN) (n = 3).

Table S1. Protein-protein BLAST analysis of *S. aureus* GntP Homology with other gram-positive bacteria.

| Representative sequence | Accession | Max Score | Total Score | Query Cover | E value | Per. ident. to  *S. aureus* | Acc. Len |
| --- | --- | --- | --- | --- | --- | --- | --- |
| *Staphylococcus aureus* | ARG47075.1 | 876 | 876 | 100% | 0 | 100 | 452 |
| *Staphylococcus schleiferi* | SUN32469.1 | 876 | 876 | 100% | 0 | 99.78 | 452 |
| *Staphylococcus caprae* | WP_002444226.1 | 795 | 795 | 100% | 0 | 89.6 | 452 |
| *Staphylococcus epidermidis* | WP_063278498.1 | 772 | 772 | 100% | 0 | 88.72 | 452 |
| *Staphylococcus saprophyticus* | BAE17557.1 | 756 | 756 | 100% | 0 | 84.96 | 452 |
| *Staphylococcus hominis* | QKH82185.1 | 754 | 754 | 99% | 0 | 86.38 | 451 |
| *Staphylococcus haemolyticus* | WP_119890362.1 | 729 | 729 | 100% | 0 | 86.06 | 452 |
| *Bacillus subtilis* | AMR64589.1 | 541 | 541 | 99% | 0 | 62.42 | 448 |
| *Enterococcus faecalis* | BDQ61214.1 | 53.5 | 53.5 | 92% | 6.00E-11 | 22.25 | 430 |

Table S2. Protein-protein BLAST analysis of *E. coli* high-affinity GntP Homology with other gram-negative bacteria.

| Representative sequence | Accession | Max Score | Total Score | Query Cover | E value | Per. ident. to  *E. coli* | Acc. Len |
| --- | --- | --- | --- | --- | --- | --- | --- |
| *Escherichia coli* (high affinity GntP) | AAN82630.1 | 857 | 857 | 100% | 0 | 100 | 438 |
| *Salmonella typhimurium* | NP_462415.1 | 842 | 842 | 100% | 0 | 97.49 | 438 |
| *Salmonella enterica* | AXC67596.1 | 838 | 838 | 100% | 0 | 97.03 | 438 |
| *Enterobacter cloacae* | QUG54440.1 | 818 | 818 | 100% | 0 | 94.75 | 438 |
| *Klebsiella pneumoniae* | XPJ82956.1 | 786 | 786 | 100% | 0 | 93.15 | 438 |
| *Pseudomonas aeruginosa* | AAD01803.1 | 365 | 365 | 98% | 6.00E-124 | 46.45 | 450 |
| *Acinetobacter baumannii* | AIL80255.1 | 362 | 362 | 100% | 3.00E-125 | 45.52 | 452 |
| *Proteus mirabilis* | - | nd | nd | nd | nd | nd | nd |

Table S3. Protein-protein BLAST analysis of *E. coli* low-affinity GntP Homology with other gram-negative bacteria.

| Representative sequence | Accession | Max Score | Total Score | Query Cover | E value | Per. ident. to  *E. coli* | Acc. Len |
| --- | --- | --- | --- | --- | --- | --- | --- |
| *Escherichia coli* (low affinity GntP) | AAN82662.1 | 875 | 875 | 100% | 0 | 100 | 446 |
| *Salmonella typhimurium* | NP_462442.1 | 858 | 858 | 100% | 0 | 97.31 | 446 |
| *Salmonella enterica* | AXC67609.1 | 853 | 853 | 100% | 0 | 96.41 | 446 |
| *Enterobacter cloacae* | QUG54454.1 | 847 | 847 | 100% | 0 | 95.96 | 446 |
| *Klebsiella pneumoniae* | XPJ82973.1 | 847 | 847 | 100% | 0 | 95.74 | 446 |
| *Proteus mirabilis* | ARX33396.1 | 655 | 655 | 100% | 0 | 76.63 | 445 |
| *Pseudomonas aeruginosa* | AAD01803.1 | 295 | 551 | 98% | 5.00E-93 | 40.78 | 450 |
| *Acinetobacter baumannii* | AIL80255.1 | 254 | 254 | 95% | 3.00E-83 | 35.6 | 452 |

Table S4. Protein-protein BLAST analysis of *S. aureus* GntP Homology with other gram-negative bacteria.

| **Representative sequence** | **Accession** | **Max Score** | **Total Score** | **Query Cover** | **E value** | Per. ident. to  ***S. aureus*** |
| --- | --- | --- | --- | --- | --- | --- |
| *Staphylococcus aureus* (GntP) | ARG47075.1 | 876 | 876 | 100% | 0 | 100 |
| *Enterobacter cloacae* (high affinity gntP) | [QUG54440.1](https://www.ncbi.nlm.nih.gov/protein/QUG54440.1?report=genbank&log$=prottop&blast_rank=1&RID=WEGBF3R3114) | 446 | 446 | 99% | 4.00E-158 | 52.77 |
| *Salmonella enterica* (high affinity gntP) | [AXC67596.1](https://www.ncbi.nlm.nih.gov/protein/AXC67596.1?report=genbank&log$=prottop&blast_rank=2&RID=WEGBF3R3114) | 443 | 443 | 99% | 3.00E-157 | 52.55 |
| *Escherichia coli* (high affinity gntP) | [AAN82630.1](https://www.ncbi.nlm.nih.gov/protein/AAN82630.1?report=genbank&log$=prottop&blast_rank=3&RID=WEGBF3R3114) | 434 | 434 | 99% | 2.00E-153 | 51.88 |
| *Salmonella typhimurium* (high affinity gntP) | [NP_462415.1](https://www.ncbi.nlm.nih.gov/protein/NP_462415.1?report=genbank&log$=prottop&blast_rank=4&RID=WEGBF3R3114) | 433 | 433 | 99% | 4.00E-153 | 52.55 |
| *Klebsiella pneumoniae* (high affinity gntP) | [XPJ82956.1](https://www.ncbi.nlm.nih.gov/protein/XPJ82956.1?report=genbank&log$=prottop&blast_rank=5&RID=WEGBF3R3114) | 427 | 427 | 99% | 6.00E-151 | 52.11 |
| *Pseudomonas aeruginosa* | AAD01803.1 | 383 | 383 | 99% | 1.00E-134 | 45.47 |
| *Acinetobacter baumannii* | [AIL80255.1](https://www.ncbi.nlm.nih.gov/protein/AIL80255.1?report=genbank&log$=prottop&blast_rank=6&RID=WEGBF3R3114) | 337 | 337 | 98% | 3.00E-115 | 43.08 |
| *Proteus mirabilis* (low affinity gntP) | [ARX33396.1](https://www.ncbi.nlm.nih.gov/protein/ARX33396.1?report=genbank&log$=prottop&blast_rank=7&RID=WEGBF3R3114) | 261 | 261 | 98% | 6.00E-86 | 35.94 |
| *Salmonella enterica* (low affinity gntP) | [AXC67609.1](https://www.ncbi.nlm.nih.gov/protein/AXC67609.1?report=genbank&log$=prottop&blast_rank=8&RID=WEGBF3R3114) | 256 | 256 | 96% | 3.00E-84 | 34.91 |
| *Salmonella typhimurium* (low affinity gntP) | [NP_462442.1](https://www.ncbi.nlm.nih.gov/protein/NP_462442.1?report=genbank&log$=prottop&blast_rank=9&RID=WEGBF3R3114) | 256 | 256 | 96% | 5.00E-84 | 35 |
| *Klebsiella pneumoniae* (low affinity gntP) | [XPJ82973.1](https://www.ncbi.nlm.nih.gov/protein/XPJ82973.1?report=genbank&log$=prottop&blast_rank=10&RID=WEGBF3R3114) | 255 | 255 | 96% | 9.00E-84 | 34.23 |
| *Enterobacter cloacae* (low affinity gntP) | [QUG54454.1](https://www.ncbi.nlm.nih.gov/protein/QUG54454.1?report=genbank&log$=prottop&blast_rank=11&RID=WEGBF3R3114) | 254 | 254 | 96% | 4.00E-83 | 34.91 |
| *Escherichia coli* (low affinity gntP) | [AAN82662.1](https://www.ncbi.nlm.nih.gov/protein/AAN82662.1?report=genbank&log$=prottop&blast_rank=12&RID=WEGBF3R3114) | 253 | 253 | 96% | 6.00E-83 | 35.36 |
| *Enterococcus faecalis* | [BDQ61214.1](https://www.ncbi.nlm.nih.gov/protein/BDQ61214.1?report=genbank&log$=prottop&blast_rank=13&RID=WEGBF3R3114) | 53.5 | 53.5 | 92% | 7.00E-11 | 22.25 |

Table S5. Bacteria strains used in this study.

| **Organism** | **Strain number** | **Source** | **Growth Media** |
| --- | --- | --- | --- |
| *Staphylococcus aureus* | *ATCC 29213* | ATCC | Luria Broth |
| *Staphylococcus epidermidis* | *ATCC 35984* | ATCC | Luria Broth |
| *Streptococcus agalactiae* | *ATCC 13813* | ATCC | Luria Broth |
| *Streptococcus pyogenes* | *ATCC 19615* | ATCC | Brain Heart Infusion Broth |
| *Enterococcus faecalis* | *ATCC 19433* | ATCC | Luria Broth |
| *Listeria monocytogenes* | *ATCC 15313* | ATCC | Luria Broth |
| *Escherichia coli* | *ATCC 25922* | ATCC | Luria Broth |
| *Klebsiella pneumoniae* | *ATCC 13883* | ATCC | Luria Broth |
| *Enterobacter cloacae* | *ATCC 7256* | ATCC | Luria Broth |
| *Acinetobacter baumannii* | *ATCC 19606* | ATCC | Luria Broth |
| *Pseudomonas aeruginosa* | *PAO1* | ATCC | Luria Broth |
| *Salmonella typhimurium* | *ATCC 29630* | ATCC | Luria Broth |
| *Proteus mirabilis* | *ATCC 29906* | ATCC | Luria Broth |
| *Staphylococcus aureus* | *JE2* | BEI resources | Luria Broth |
| *Staphylococcus aureus* | *Transposon Mutant NE952* | BEI resources | Luria Broth |
| *Staphylococcus aureus* | *Transposon Mutant*  *NE1124* | BEI resources | Luria Broth |
| *Escherichia coli Nissle 1917* |  | Creative Biolab | Luria Broth |

Table S6. Clinical strains of bacteria used in this study.

| **Organism** | **Strain number** | **Source** | **Growth Media** |
| --- | --- | --- | --- |
| *MSSA #1* | *ATCC 29213* | BEI resources | Luria Broth |
| *MSSA #2* | *ATCC 35984* | BEI resources | Luria Broth |
| *MSSA #3* | *ATCC 13813* | BEI resources | Luria Broth |
| *MSSA #4* | *ATCC 19615* | BEI resources | Luria Broth |
| *MSSA #5* | *ATCC 19433* | BEI resources | Luria Broth |
| *MSSA #6* | *ATCC 15313* | BEI resources | Luria Broth |
| *MRSA #1* | *ATCC 25922* | BEI resources | Luria Broth |
| *MRSA #2* | *ATCC 13883* | BEI resources | Luria Broth |
| *MRSA #3* | *ATCC 7256* | BEI resources | Luria Broth |
| *MRSA #4* | *ATCC 19606* | BEI resources | Luria Broth |
| *MRSA #5* | *ATCC 10145* | BEI resources | Luria Broth |
| *MRSA #6* | *ATCC 29630* | BEI resources | Luria Broth |
| *S. epidermidis #1* | *HIP04645* | BEI resources | Luria Broth |
| *S. epidermidis #2* | HIP4680 | BEI resources | Luria Broth |
| *S. epidermidis #3* | 12333 | BEI resources | Luria Broth |
| *S. epidermidis #4* | NRS34 | BEI resources | Luria Broth |
| *S. epidermidis #5* | W25756 | BEI resources | Luria Broth |
| *S. epidermidis #6* | 01-004-2919 | BEI resources | Luria Broth |
| *E. faecalis #1* | HH22 | BEI resources | Luria Broth |
| *E. faecalis #2* | CH19 | BEI resources | Luria Broth |
| *E. faecalis #3* | B4008 | BEI resources | Luria Broth |
| *E. faecalis #4* | V587 | BEI resources | Luria Broth |
| *E. faecalis #5* | B3196 | BEI resources | Luria Broth |
| *E. faecalis #6* | B3286 | BEI resources | Luria Broth |
| *E. coli #1* | 1093925 | BEI resources | Luria Broth |
| *E. coli #2* | 1099705 | BEI resources | Luria Broth |
| *E. coli #3* | 1121580 | BEI resources | Luria Broth |
| *E. coli #4* | 1144993 | BEI resources | Luria Broth |
| *E. coli #5* | 867285 | BEI resources | Luria Broth |
| *E. coli #6* | 959900 | BEI resources | Luria Broth |
| *S. typhimurium #1* | S5682 | BEI resources | Luria Broth |
| *S. typhimurium #2* | S9328 | BEI resources | Luria Broth |
| *S. typhimurium #3* | S5564 | BEI resources | Luria Broth |
| *S. typhimurium #4* | S8934 | BEI resources | Luria Broth |
| *S. typhimurium #5* | ST2380 | BEI resources | Luria Broth |
| *S. typhimurium #6* | ST2488 | BEI resources | Luria Broth |
| *K. pneumoniae #1* | UCI 41 | BEI resources | Luria Broth |
| *K. pneumoniae #2* | UCI 42 | BEI resources | Luria Broth |
| *K. pneumoniae #3* | CHS 61 | BEI resources | Luria Broth |
| *K. pneumoniae #4* | CHS 62 | BEI resources | Luria Broth |
| *K. pneumoniae #5* | CHS 63 | BEI resources | Luria Broth |
| *K. pneumoniae #6* | 1040205 | BEI resources | Luria Broth |
| *A. Baumannii #1* | MRSN 7735 | BEI resources | Luria Broth |
| *A. Baumannii #2* | MRSN 24008 | BEI resources | Luria Broth |
| *A. Baumannii #3* | MRSN 30945 | BEI resources | Luria Broth |
| *A. Baumannii #4* | MRSN 7576 | BEI resources | Luria Broth |
| *A. Baumannii #5* | MRSN 10372 | BEI resources | Luria Broth |
| *A. Baumannii #6* | MRSN 7460 | BEI resources | Luria Broth |

Table S7. Estimated absorbed dose (mGy/MBq) data extrapolated from female mice data using ICRP60.

| **Absorbed Dose (mGy/MBq) or Equivalent Dose (mSv/MBq)** | Adult female (60 kg) |  |  |
| --- | --- | --- | --- |
| **Organ** |  |  |  |
| Adrenals | 0.0057 | ± | 0.0001 |
| Brain | 0.0014 | ± | 0.0000 |
| Breasts | 0.0039 | ± | 0.0001 |
| Gallbladder Wall | 0.0055 | ± | 0.0001 |
| LLI Wall | 0.0109 | ± | 0.0005 |
| Small Intestine | 0.0073 | ± | 0.0001 |
| Stomach Wall | 0.0054 | ± | 0.0001 |
| ULI Wall | 0.0071 | ± | 0.0000 |
| Heart Wall | 0.0057 | ± | 0.0001 |
| Kidneys | 0.0155 | ± | 0.0010 |
| Liver | 0.0036 | ± | 0.0001 |
| Lungs | 0.0072 | ± | 0.0002 |
| Muscle | 0.0057 | ± | 0.0000 |
| Ovaries | 0.0107 | ± | 0.0004 |
| Pancreas | 0.0058 | ± | 0.0001 |
| Red Marrow | 0.0052 | ± | 0.0000 |
| Osteogenic Cells | 0.0078 | ± | 0.0002 |
| Skin | 0.0040 | ± | 0.0001 |
| Spleen | 0.0055 | ± | 0.0001 |
| Thymus | 0.0048 | ± | 0.0001 |
| Thyroid | 0.0043 | ± | 0.0001 |
| Urinary Bladder Wall | 0.2138 | ± | 0.0230 |
| Uterus | 0.0162 | ± | 0.0011 |
| Total Body | 0.0058 | ± | 0.0000 |
| **Effective Dose (mSv/MBq)** | 0.0174 | ± | 0.0013 |

Table S8. Estimated absorbed dose (mGy/MBq) data extrapolated from male mice data using ICRP60.

| **Absorbed Dose (mGy/MBq) or Equivalent Dose (mSv/MBq)** | Adult male (73 kg) |  |  |
| --- | --- | --- | --- |
| **Organ** |  |  |  |
| Adrenals | 0.0051 | ± | 0.0013 |
| Brain | 0.0012 | ± | 0.0002 |
| Breasts | 0.0031 | ± | 0.0005 |
| Gallbladder Wall | 0.0050 | ± | 0.0009 |
| LLI Wall | 0.0094 | ± | 0.0007 |
| Small Intestine | 0.0065 | ± | 0.0003 |
| Stomach Wall | 0.0046 | ± | 0.0008 |
| ULI Wall | 0.0059 | ± | 0.0004 |
| Heart Wall | 0.0050 | ± | 0.0009 |
| Kidneys | 0.0331 | ± | 0.0225 |
| Liver | 0.0037 | ± | 0.0007 |
| Lungs | 0.0065 | ± | 0.0012 |
| Muscle | 0.0049 | ± | 0.0003 |
| Pancreas | 0.0051 | ± | 0.0011 |
| Red Marrow | 0.0044 | ± | 0.0005 |
| Osteogenic Cells | 0.0060 | ± | 0.0008 |
| Skin | 0.0033 | ± | 0.0004 |
| Spleen | 0.0049 | ± | 0.0012 |
| Testes | 0.0069 | ± | 0.0004 |
| Thymus | 0.0038 | ± | 0.0006 |
| Thyroid | 0.0036 | ± | 0.0006 |
| Urinary Bladder Wall | 0.1855 | ± | 0.0468 |
| Total Body | 0.0050 | ± | 0.0004 |
| **Effective Dose (mSv/MBq)** | 0.0151 | ± | 0.0021 |

**Materials and Methods**

All reagents and solvents were purchased from Sigma-Aldrich and used without further purification. Nuclear magnetic resonance (NMR) spectra were acquired using a Bruker Advance III HD 400 Hz instrument at the UCSF Nuclear Magnetic Resonance Laboratory. Mass spectrometry was performed at the University of California, Berkeley Spectrometry Facility. [^18^F]FDG was generated in the UCSF radiopharmaceutical facility. Radioactivity was measured by using a Hidex Automatic Gamma Counter (Hydex Oy). μPET/CT imaging was conducted using a nanoScan μPET/CT (Mediso). All protocols were approved by the University of California, San Francisco Radiation Safety, Biosafety, and Animal Care and Use Committees. [^18^F]FDM was synthesized according to our established protocol [12].

**Synthesis of FGA and [^18^F]FGA**

Non-radioactive FGA was synthesized via glucose oxidase (5 mg) mediated oxidation of FDG (50 mg) in 50 mM sodium citrate buffer (1 mL, pH 6.0) at 37°C for 4 hours. Similarly, radiosynthesis of [^18^F]FGA was conducted using clinical [^18^F]FDG without further purification. An [^18^F]FDG solution (typically 0.5 – 2.0 GBq in 0.5 mL) was added to a vial containing glucose oxidase (1 mg). The reaction was incubated at 35°C for 30 min. Both non-radioactive FGA and [^18^F]FGA were purified by simple cartridge-based purification. The enzyme was precipitated by the addition of 0.5 mL of water and 2.0 mL of MeCN, followed by centrifugation at 13,200 rpm for 5 min. The supernatant was diluted with MeCN (17 mL) and passed through a NH_2_ Plus Sep Pak cartridge. Residual FDG or [^18^F]FDG was washed with 20 mL of 95% MeCN/water, according to our established protocol. After drying using an NH_2_ Sep Pak, an alumina light Sep Pak was connected in series. The product was eluted with 3 mL of saline for FGA or [^18^F]FGA. The chemical identity and purity of FGA were confirmed by NMR and mass spectrometry. The radiochemical purity of [^18^F]FGA was confirmed by radio-HPLC.

**2-deoxy-2-fluoro-gluconic acid (FGA)**

White solid (17.4 mg, 32%). ^1^H NMR (400 MHz, D_2_O) δ 4.87 (dd, *J* = 50.0, 3.7 Hz, 1H, C2), 4.09 (dt, *J* = 24.4, 3.3 Hz, 1H, C3), 3.81 – 3.73 (m, 1H, C6), 3.73 – 3.65 (m, 2H, C4 and C5), 3.64 – 3.54 (m, 1H, C6’); ^13^C NMR (100 MHz, D_2_O) δ 175.22 (d, *J* = 21.3 Hz, C1), 92.18 (d, *J* = 185.5 Hz, C2), 71.50 (d, *J* = 3.1 Hz, C4), 71.12 (C5), 70.40 (d, *J* = 18.8 Hz, C3), 62.67 (C6); ^19^F NMR (376 MHz, D_2_O) δ -196.68. MS (EI-Neg) m/z [M-H]^-^ calcd for C_6_H_10_FO_6_^-^ 197.0466, found 197.0467.

***In vitro* study**

*In vitro* assays were performed according to our established protocol [55]. Briefly, bacterial cultures were incubated with ^18^F-tracers (final concentration: 0.1 MBq/mL) at 37 °C for 90 min. Uptake was determined by measuring the radioactivity in the pellets and supernatants using a gamma counter. For inhibition assays, *S. aureus* cultures were incubated with [^18^F]FGA and inhibitors (1 mM of GA or FGA, and 0.1 mM of PCMB) at 37 °C for 90 min.

**Animal models**

All animal studies were approved by the Institutional Animal Care and Use Committee at UCSF and performed following UCSF guidelines. Female CBA/J mice (9 to 11 weeks) were used for all experiments unless otherwise specified. The mice used in this study were not immunosuppressed. The LPS-induced myositis model was generated by intramuscular injection of lipopolysaccharide (LPS, 5 mg/kg) into the left triceps muscle 96 h before PET/CT imaging study. The bacterial myositis model was generated by intramuscular injection of 1 × 10^6^ CFUs live or 1 × 10^7^ CFUs heat-killed bacteria (50 μL) into the left and right triceps muscles, respectively, 6 h before the imaging study. For the *S. aureus* transposon mutant myositis model, mice (n = 4 for each) were inoculated intramuscularly with 1 × 10^6^ CFUs of *ΔgntP* mutant and JE2 *S. aureus* strains into the left and right triceps muscles, respectively, 6 h before the imaging study. For the pulmonary infection model, mice (n = 4) were inoculated intratracheally with *K. pneumoniae* (1 × 10^6^ CFUs in 100 μL) 24 h before the imaging study.

**Imaging data acquisition**

All imaging data were acquired using a small animal PET/CT scanner (nanoScan, Mediso). [^18^F]FGA (~ 8 MBq, 100 uL saline) was administered intravenously via tail vein for imaging studies. Mice were anesthetized with 2% isoflurane/oxygen during data acquisition. List-mode data were acquired to generate 53 consecutive dynamic frames (2s × 15, 5s × 6, 10s × 6, 30s × 4, 60s × 6, 300s × 16 frames) for reconstruction of volumes. For imaging uninfected mice (conscious condition), a static scan (5 min) was performed 85 min after tracer administration. For imaging infected or sterile inflammation mice, static scans (10 min) were conducted 80 min after tracer administration. Image reconstruction was performed using a vendor-provided iterative algorithm. CT-based attenuation correction and scatter correction were performed, and the pre-calculated quantification calibration factor was used to convert the raw reconstructed pixel value to a physical unit of Bq/ml. Volumes of interest were manually drawn for brain, lungs, liver, kidneys, and urinary bladder in Amide software to generate organ time-activity curves (17). The radioactivity in blood pool was estimated from the left atrium.

**Radiation dose estimation**

Volumes of interest (VOIs) were drawn on co-registered PET and CT images for the brain, lungs, heart, liver, kidneys, and urinary bladder. All VOIs were elliptical cylinder (5 mm long axis, 3 mm short axis, and 5 mm height for the brain), cylinder (3 mm diameter and 3 mm height for the kidneys), or spheres (3 mm diameter for lungs, liver, and urinary bladder, and 4 mm diameter for heart), and they were placed well within the anatomical boundaries to minimize spill-over or spill-in of radioactivity. The mean values (in Bq/ml) in these VOIs were multiplied by standard mouse organ volumes (in mL) to estimate total activity (in Bq) within these organs except for the urinary bladder. Since the bladder accumulated a large amount of radioactivity very quickly for this radiotracer, an attempt was made to capture the whole bladder activity for individual animal, instead. The total activity within the entire animal subtracted by all organ activities was used as activity in the remainder of the body. The percent of injected activity within the defined organs (%IA) was extrapolated to human-equivalent values using ratios of standard human organ weights to mouse organ weights, again except for the urinary bladder. %IA for the bladder was estimated for individual animal, and the human-equivalent value was assumed to be the same.

These input %IA data for each organ and the remainder of the body were curve-fitted to derive time-integrated activity coefficients (TIACs, also known as residence times) (in Bq-hr/Bq) and organ absorbed and equivalent doses and whole-body effective dose for human equivalents were estimated for each animal data. The data from these animals were averaged to derive absorbed and equivalent organs doses (in mGy/MBq or mSv/MBq), and whole-body effective dose (in mSv/MBq). Using TIACs as input, dose estimations were performed using OLINDA version 1.1 using ICRP Publication 60 tissue weighting factors as well as OLINDA version 2.0 using ICRP Publication 103 tissue weighting factors.

**Statistical analysis**

All statistical analyses and graph generation were performed using GraphPad Prism v10 software (GraphPad Software Inc.). Statistical significance was performed using ANOVA or Student’s *t*-tests depending on the study design. *P* values less than 0.05 were considered statistically significant. All data were expressed as the mean ± standard deviations (SD), except TACs which were expressed as the mean ± SEM.
